# 3D Guided Dental Implant Placement: Impact on Surgical Accuracy and Collateral Damage to the Inferior Alveolar Nerve

**DOI:** 10.3390/dj9090099

**Published:** 2021-09-02

**Authors:** Amit Mistry, Cemal Ucer, John D. Thompson, Rabia Sannam Khan, Emina Karahmet, Farooq Sher

**Affiliations:** 1 Amit Mistry Implants, The Whitehouse, Greenalls Avenue, Warrington WA4 6HL, UK; implantsbyam@gmail.com; 2 ICE Postgraduate Dental Institute and Hospital, Salford M50 3XZ, UK; cemalucer@me.com; 3 School of Health and Society, University of Salford, Salford M5 4WT, UK; j.d.thompson@salford.ac.uk; 4 Education and Research Director, ICE Postgraduate Dental Institute and Hospital, 24 Furness Quay, Salford M50 3XZ, UK; r.s.khan@salford.ac.uk; 5 Department of Medical Chemistry, Faculty of Pharmacy, University of Tuzla, 75000 Tuzla, Bosnia and Herzegovina; emina.karahmet@gmail.com; 6 Department of Engineering, School of Science and Technology, Nottingham Trent University, Nottingham NG11 8NS, UK

**Keywords:** sustainable materials, dental implants, inferior alveolar nerve (IAN), free hand surgery, fully guided surgery and surgical precision

## Abstract

An increase in the number of implants placed has led to a corresponding increase in the number of complications reported. The complications can vary from restorative complications due to poor placement to damage to collateral structures such as nerves and adjacent teeth. A large majority of these complications can be avoided if the implant has been placed accurately in the optimal position. Therefore, the aim of this in vitro pilot study was to investigate the effect of freehand (FH) and fully guided (FG) surgery on the accuracy of implants placed in close proximity to vital structures such as the inferior alveolar nerve (IAN). Cone-beam computed tomography (CBCT) and intraoral scans of six patients who have had previous dental implants in the posterior mandible were used in this study. The ideal implant position was planned. FG surgical guides were manufactured for each case. In this study, the three-dimensional 3D printed resin models of each of the cases were produced and the implants placed using FG and FH methods on the respective models. The outcome variables of the study, angular deviations were calculated and the distance to the IAN was measured. The mean deviations for the planned position observed were 1.10 mm coronally, 1.88 mm apically with up to 6.3 degrees’ angular deviation for FH surgery. For FG surgical technique the mean deviation was found to be at 0.35 mm coronally, 0.43 mm apically with 0.78 degrees angularly respectively. The maximum deviation from the planned position for the apex of the implant to the IAN was 2.55 mm using FH and 0.63 mm FG. This bench study, within its limitations, demonstrated surgically acceptable accuracy for both FH and FG techniques that would allow safe placement of implants to vital structures such as the IAN when a safety zone of 3 mm is allowed. Nevertheless, a better margin of error was observed for FG surgery with respect to the angular deviation and controlling the distance of the implant to the IAN using R2 Gate^®^ system.

## 1. Introduction

Implant surgery has become a standard procedure for the rehabilitation of dental function due to edentulism, trauma or ablative surgery. With increasing demand, different approaches have been proposed for the treatment of complex implant cases [1]. There is, however, consensus that an increase in the number of implants placed has led to a corresponding increase in the number of complications reported. The complications can vary from restorative problems due to implant positional errors to collateral damage to vital structures such as nerves and adjacent teeth. Significant morbidity including chronic neuropathic pain leading to suicidal thoughts has been reported due to iatrogenic nerve damage following dental implant surgery [2]. A large majority of these mechanical, technical and biological complications can be avoided if implant placement is planned and executed with better surgical accuracy and precision [3].

Developments in cone-beam computed tomography (CBCT) and intraoral scanning have facilitated the use of digital implant workflow (DWF). The ideal functional and restorative positions of teeth can be planned digitally for the insertion of implants accurately in three dimensions to achieve the desired outcome. The use of digital workflow in oral and maxillofacial surgery is expanding in the areas of performing osteotomies, zygomatic implants, bone regeneration, orthognathic surgeries where greater surgical precision and accuracy is essential [4]. To satisfy the high expectations of patients and to ensure an adequate and predictable long-term outcome, implant treatment requires prosthodontically guided, three-dimensional assessment and planning [5]. DWF can optimize the process, as they provide valuable diagnostic information and facilitate backward planning to improve safety and efficiency, which contribute to a more predictable outcome.

However, inherent manufacturing inaccuracies and human error must be recognized and managed appropriately to avoid collateral damage to vital structures [6]. Optimal positioning ensures adequate bone volume surrounding the implant with correct load distribution. Whilst freehand (FH) implant placement has been the standard approach [5,6], the surgical accuracy of this method can be limited. Despite the use of anatomical landmarks or stents, FH surgery relies on good three-dimensional (3D) spatial awareness and high levels of surgical experience to place the dental implant correctly within the limited volume of residual bone [7,8]. A successful outcome, therefore, relies on individual operator experience and would be impossible to standardize. It is believed that FG implant surgery, which uses virtual 3D planning, has the potential to allow implant positioning with improved accuracy, safety, and efficiency.

A recent systematic review showed that the five-year survival rates of implants placed using digitally-designed static surgical guides are comparable to the estimated overall survival rate (95.6% over five years), despite the complex nature of the treatments done with guided surgery [6]. However, the surgical accuracy and precision of implants placed with FG have not yet been fully established. FG surgery involves using a 3D printed guide incorporating a series of drilling tubes to match the increasing drill size when preparing the implant osteotomy [9,10]. The implant is finally placed through the guide to replicate the virtually planned position. Younes et al. [11] found this technique to be the most accurate method of implant placement. They demonstrated mean coronal global deviation (CGD) of 0.73 mm, apical global deviation (AGD) 0.97 mm and angular deviation (AD) 2.30 degrees respectively compared with the planned implant position and suggested that FG surgery should be regarded as the gold standard technique for implant placement.

Therefore, the implant placement accuracy is necessary and can be achieved using anatomical landmarks and basic surgical stents, achieve optimum aesthetics and avoid the collateral damage of vital structures such as adjacent teeth roots and nerves. The consequence of inaccurate implant placement can cause an increased risk of peri-implantitis due to unfavourable prosthetics and loading of the implants [7]. Furthermore, damage to the IAN is significant, debilitating and life-changing for the patient. The IAN can be identified quantitatively on R2 Gate^®^ software using Hounsfield units (HU) [8]. Therefore, it can be differentiated from the adjacent bone as opposed to the subjective mapping on the cone-beam computed tomography (CBCT) by a clinician. A safety zone of 2 mm has been proposed by consensus [9,10] to avoid damaging the inferior alveolar nerve (IAN). This may be inadequate as discussed by Renton (2010) due to the apex of the drill being 1.5 mm longer than the implant [2]. Greenstein and Tarnow (2006) previously proposed a safety zone of 4 mm to account for the additional drill length. This allows planning of the implant placement to be so precise that it can avoid damage to the IAN [11].

This study aimed to investigate if FG surgery can be used to place dental implants with a higher degree of surgical accuracy that would be necessary to reduce the risk of collateral damage occurring to nearby vital structures such as the IAN when compared with FH surgery. It is believed that FG implant surgery, which uses virtual 3D planning, has the potential to allow implant positioning with improved accuracy, safety, and efficiency. FG surgery involves using a 3D printed guide incorporating a series of drilling tubes to match the increasing drill size when preparing the implant osteotomy [12,13]. Therefore, in this study, the implant was finally placed through the guide to replicate the virtually planned position to analyse the surgical accuracy of the technique [14].

## 2. Materials and Methods

This bench study was designed to use a fully digital workflow to virtually plan the placement of dental implants into stereolithographic models of mandibles to investigate the accuracy of the FG 3D implant placement technique compared with free-hand drilling. Ethical approval was obtained from the University of Salford Ethics Committee (HST1920-007).

### 2.1. Eligibility Criteria for the Selection of Cases

In this study, the CBCT images of six patients at ICE Postgraduate Institute and Hospital were used to create stereolithographic models for implant placement. Inclusion and exclusion criteria were as follows: The inclusion criteria were based on selecting the patients with premolar/molar region of the mandible in their mouth. Adequate bone height between the alveolar ridge and inferior alveolar nerve (IAN) for a 4 mm safety zone for a 10 mm implant. Patients, in which implants can be placed 1.5 mm from adjacent teeth and where the clearance of 1.5 mm between the shoulder of implant and buccal bone. The exclusion criteria were based on patients where it was not able to trace the full course of the IAN canal and/or mental foramina by a practitioner. Additionally, with other incidental findings or pathology on CBCT. The planned procedure was to place a single dental implant retained crown using a (Megagen AnyRidge^®^ (Bedfordshire, UK)) 4.0 × 10 mm implant in the correct restorative position with the adequate bone surrounding the implant for long-term stability. A summary of cases is described below in Table 1. For each case, the corresponding. STL files and CBCT data needed to be uploaded to the R2 gate, therefore, 3D planning for each case was required and the following protocols were followed to achieve it.

### 2.2. 3D Protocol Planning

**Figure 1:**
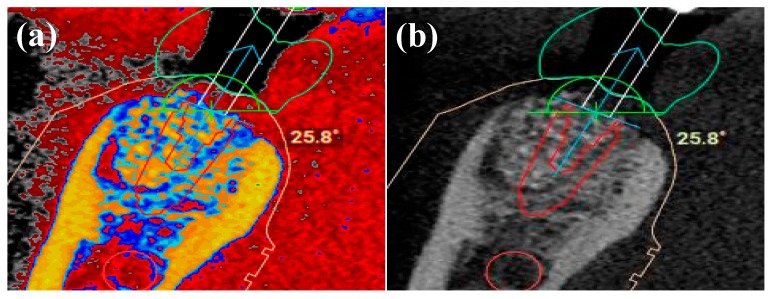
(**a**) Different HU values were used to create a colour map and (**b**) to identify different structures. Bone is represented as yellow and soft tissue as red. This allowed the inferior alveolar nerve IAN to be traced accurately.

MORITA (Veraviewepocs) CBCT unit was used to acquire sectional images with a 5 × 5 cm field of view (FOV) of the mandible of patients undergoing routine dental implant treatment at the hospital. STL files were obtained from intraoral optical scans (IOS) as a digital impression of the surrounding anatomy of the teeth and the soft tissues using Trios/3Shape^®^ unit (Straumann, UK). The STL images were merged with the CBCT data and the IAN was traced using Megagen R2Gate^®^ planning software (www.r2gate.com (accessed on 10 May 2021)). Hounsfield Units (HU) values for bone (dense bone > 1250 HU for D1 to 350–150 HU for trabecular bone) were used to identify the course of the IAN. R2Gate uses Digital-EYE to delineate bone of different densities according to HU values; 256 shaded of grey are converted to a colour map of bone morphology to help plan the optimal drilling sequence (Figure 1). Once the virtual planning was verified, two models for each case were 3D printed from the STL files using Envision TEC printer (Vida, Dental 3D Printers, Dearborn, MI, USA) [10].

**Figure 2:**
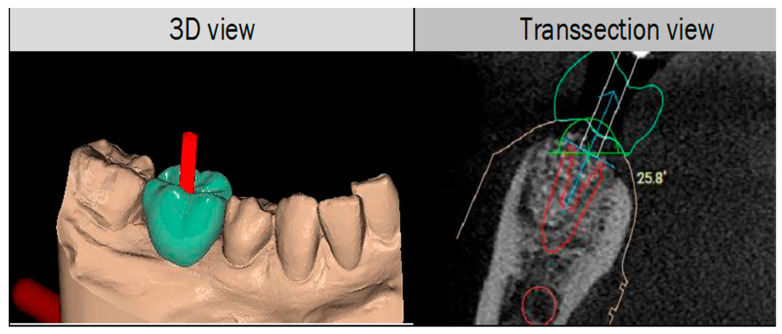
The location of the IAN, the safety zone and planned implant position guided by the virtually designed restoration using Megagen R2 Gate.

The printer can print to an accuracy of 30 microns using e-guide tint resin to ensure consistency in the manufacturing of the models [15]. A suitable implant was selected from the R2Gate^®^ digital library and placed in close proximity to the location of the IAN and MN in a restoratively driven position as dictated by the virtually generated tooth. For all cases, a (Megagen AnyRidge^®^) implant (4.0 mm diameter by 10 mm length) was virtually planned with the following parameters (Figure 2). A minimum “safety zone” of 4 millimetres to the IAN was allowed according to the current consensus [16]. A length of 1.5 millimetres from the implant of the adjacent teeth was in line with clinical guidelines [17]. A clearance of 1.5 millimetres was planned between the shoulder of the implant and the buccal bone plate according to established clinical guidelines [18]. The distance, between the apex of the implant and the IAN of the virtually planned implant, were measured in a perpendicular slice through the apex of the planned implant using the measuring tool of the software. The distance is the shortest point from the apex of the implant to the IAN. The virtual plan was checked and verified by the researcher as well as the technician.

### 2.3. In-Vitro Implant Placement

An experienced implant dentist who has placed over 50 implants has placed the implants on the models. As shown by Renouard, Amalberti and Renouard et al. [19], this level of experience is needed to ensure the accuracy of implant placement. For each of the clinical cases, the implants were being placed in the 3D printed replica models using (Megagen AnyRidge^®^) freehand surgical kit and using the Megagen^®^ universal fully guided kit on a duplicate 3D printed model produced as outlined above. The implants were placed using sterile water irrigation to avoid overheating of the resin on the model which could affect the accuracy of the implant placement. For the FH placement, the virtually planned position of the implant was available to enable the clinician to place the implant as close to the planned position as possible. The FH implants were placed 2 mm deeper on the drill markings to take into account the depth of the soft tissue present [20]. The Megagen AnyRidge^®^ drill protocols were used to prepare the implant sites according to FH/FG placements. The model was rinsed with water after drilling to allow removal of any debris in the osteotomy site which could affect the implant seating.

### 2.4. Digital Scanning of the Implants Using IOS

**Figure 3:**
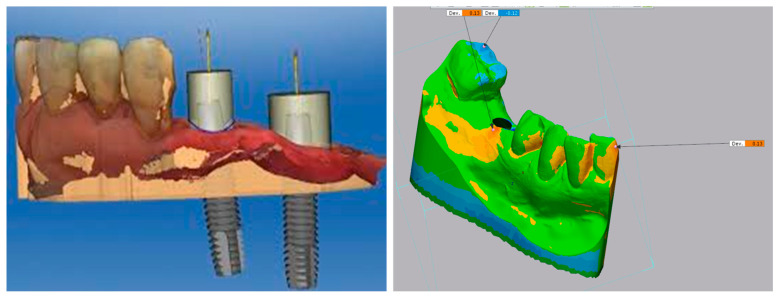
**A** 3D printed STL showing a difference of less than 0.13 mm of model accuracy of the planned position in FH surgical technique.

Once the implants were placed in the printed models, (Megagen AnyRidge^®^) scan flags were attached to the implants. The model with the scan flags was scanned using a laboratory-based scanner (need specs of the scanner) to create an STL file of the model with a scan flag attached (Figure 3).

### 2.5. Scan Analysis Using EXOCAD^®^

These STL files of the scanned implants were imported into Exocad dental laboratory software and digital (Megagen AnyRidge^®^) 4.0 × 10 mm implants were virtually fitted onto the scan flag to image the digital position of the implant that has been placed in the model. These STL files were then exported out of EXOCAD^®^ and imported into the R2 Gate^®^ virtual planning software for compression with the planned implant positions and data analysis [21,22].

### 2.6. Measurement of Deviations in Implant Position

The STL file of the placed implant was overlayed on the original. The STL file of the planned implant placement used Geomagic Control software. This software has been used in many similar implant placement comparison studies and was found to be reliable [23]. The Geomagic software was also be used to verify the accuracy of the model that has been overlaid so that any deviation detected is because of implant placement as opposed to any inaccuracies in the model manufacture process.

### 2.7. Data Analysis

Data were recorded and descriptive statistics were produced using Microsoft Excel. The data were considered paired as the measurements for free-hand and fully guided surgery came from the same sample of patients [24]. The neck of the implant was considered a consistent point of entry and was maintained for FG and FH surgical techniques. As such, paired t-tests were completed for the following outcomes comparing free-hand and fully guided: coronal midpoint deviation, apical midpoint deviation, and vector angle deviation; in addition, the freehand and fully guided distance from the IAN was compared to the planned position. Test alpha for significance was set at 0.05 for all of the above tests.

## 3. Results and Discussion

In comparison to FH and FG techniques using the stereolithographic models to the planned implant position using Geomagic Control Software (3D systems Corporation, Cary, NC, USA) the summary of the deviation to the planned position is identified for free-hand (Table 2) and fully guided (Table 3) for all cases is shown below.

**Figure 4:**
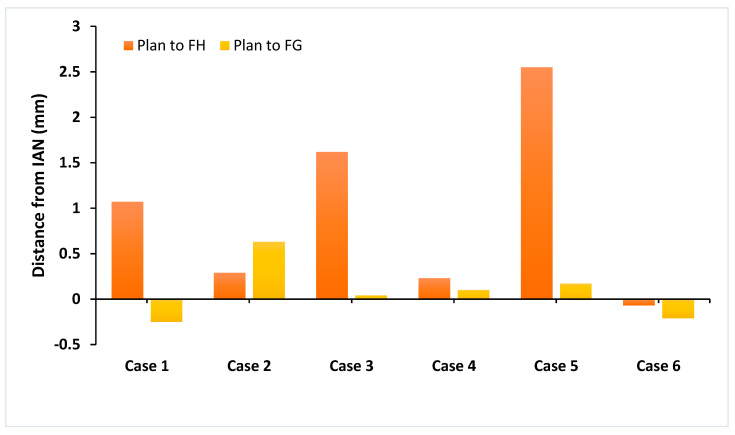
Indicates the deviation from the planned implant position and distance from inferior alveolar nerve (IAN). Negative values indicate a final position closer to the IAN than planned.

The comparison of the planned position to the placed position for FH and FG techniques can be seen in Figure 4. The mean position of the FG technique (0.08 ± 0.32 mm) was closer to the planned position than the FH technique (0.97 ± 1.00 mm). There was a general tendency for the FH technique to leave a larger safety zone to the IAN and only in Case 6 was the free-hand position closer to the IAN than planned [25]. The fully guided placement was also closer to the IAN than planned in Case 6 and Case 1. The guided approach had much greater consistency, represented by a lower standard deviation, but overall, the comparison of fully guided and free-hand techniques in terms of distance of the apex to the IAN was not significantly different from the planned position (*p* = 0.10).

However, an FG technique was found to achieve a position closer to the planned position for all other measurements in all cases. For the measured vector angle the fully guided technique was found to be significantly better (*p* = 0.01), with a much smaller mean deviation (0.78 ± 0.33 mm versus 6.43 ± 3.82 mm). For the measured apical deviation, the fully-guided technique was significantly better (*p* = 0.02), with a much smaller mean deviation (0.43 ± 0.27 mm versus 1.88 ± 0.95 mm). For the measured coronal variation, there was no significant difference between the two techniques (*p* = 0.05), but again there was a smaller mean deviation for the fully guided technique (0.35 ± 0.24 mm versus 1.10 ± 0.57 mm) [26].

Dental implant placement can be subject to variation inaccuracy due to operator experience and the variety of techniques and kits available to the implant surgeon [27]. In this bench study, using a single experienced operator, there was no statistically significant difference in the distance of the apex to the IAN between the FH and FG and the planned position (*p* = 0.10). Nevertheless, within the limitations of this study, 3D FG surgery produced an outcome closer to a planned position particularly concerning angular deviation- a finding that should give the operator confidence that they can avoid damage to vital structures close to the site of surgery. However, the tolerance of FG system can be affected by several possible sources of error including 3D printing, post-processing, CBCT acquisition and resolution, positional errors, tube to bone distance. These factors should be taken into consideration when planning implants close to the IAN or other vital structures [26,28].

Comparison to recent literature (Table 4) indicated that FG implant placement could be more accurate than FH implant placement concerning 3D planned position. It is encouraging that sub-millimetre deviation from the planned entry (range 0.4–1.4 mm) and position of the apex (range 0.4–1.6 mm) has been demonstrated with FG surgery which is consistent with the results of the current study except for Tahmaseb et al. [29] which was a systematic review, all are patient studies using a single operator where the number of patients ranged from 11–59 years of age. Generally, the range of angular deviation is greater for both FH (6.4–9.1°) and FG (0.8–5.0°) techniques. This contrasts with the current bench study which showed less than 2° mean angular deviation when FG placements were compared with FH placements. Tahmaseb et al., which was a systematic review, all are patient studies using a single operator where the number of patients ranged from 11–59 years of age [29].

The higher accuracy observed between 3D planned and actual implant positions in this study, for both FH and FG techniques, may be due to several factors including the use of stereolithographic models for implant placement rather than real patient cases, use of innovative R2 Gate^®^ FG technique, implant characteristics such as the design, diameter and length of the implant, operator skills, and type of the surgical guide (tooth-supported). Our results showed the accuracy of FH placement to be acceptable and a viable alternative to FG surgery but the angular deviation of the free-hand technique in Case 2 (9.12°) would have resulted in perforation of the lingual cortex, with the risk of collateral damage occurring to an adjacent vital structure [34]. On the other hand, when FG was performed in Case 2, the angular deviation was reduced to 0.97°, thus mitigating this risk. A similar trend was also observed with Case 5, with a reduction in angular deviation from 11.7° to 0.97°.

The mean deviation from the planned and actual implant positions was smaller with FG surgery compared with FH. This would be expected since FH is more subjective, relying on anatomical landmarks as a reference point. However, it is interesting to note that in two of the cases the implant placed using the FG technique was closer to the IAN than the planned position for a fraction of a millimetre (0.21 mm and 0.25 mm respectively). The mean 3D deviations seen in this study were less than those observed in previous studies This could be explained because the current study used tooth-supported guides in a bound saddle configuration with teeth on either side of the planned implant placement. This improves the positioning and stability of the guide compared with a single bound saddle or mucosa supported guides, such as that used by Vercruyssen et al. [32].

In future, it will be significant to control the angular deviation while placing implants to avoid vital structures. These structures include the sinus floor anatomy, adjacent teeth roots and the mandibular nerve and the anterior loop of the IAN. It was noticed in one of the present study cases, the implant placement by FH surgical technique is nearly perforated the lingual cortex as a result of an angular deviation of 9.121° compared to the planned implant position. The perforation could lead to perforation of the branch of the lingual artery and can cause a fatal bleed [35]. While FG surgery was utilized in the same case, the angular deviation was reduced to 0.972° which was more than adequate to prevent any perforation of the lingual cortex and reduced the risk of damaging the lingual artery.

The precision of FG surgery to reduce the angular deviation was seen in one case (6), where the planned position of the apex of the implant was lateral to the IAN and the distance is only 1.29 mm. Although in our other cases (1 and 2) the similar criteria were not met due to an in vivo study as the planned distance to the IAN is only 1.29 mm from the apex position of the implant, it was included due to the fact as this is a laboratory-based experiment and there would be no consequence of damage to the IAN or other vital structures. The FH surgery for Case 6 showed a reduction in the distance to the IAN compared to the planned outcome and the apical portion of the implant ended up more medial to the IAN solely by chance. With FG surgery the implant was closer to the IAN by 0.21 mm and in a clinical situation, the IAN would have been damaged by the 1.5 mm extension of the cutting tip of the drill which would directly damage the nerve.

A crushing injury to the IAN would have also occurred and this could have caused by the apical portion of the implant compressing the bony housing of the superior aspect of the IAN canal [36]. This could lead to the patient experiencing signs and symptoms of IAN injury. Crushing injuries are not very common in implant dentistry and there are ways to reduce the incidence of this occurring. Therefore, local anaesthetic given as a buccal and lingual infiltration as opposed to a regional inferior dental block (IDB) is preferred in crushing injuries. By using infiltrations only, the IAN can still feel the sensation of pressure and pain so when the implant is closer to the IAN the patient could feel the pressure. In that situation, the patient needs to be instructed to make the clinician aware of any pressure so that implant insertion can be stopped and to stop further crushing of IAN. The clinician can reverse the implant to relieve any pressure and allow the peripheral nerve a chance of regeneration [37]. An alternative method could be by attaching depth stop to the implant drills and the implant driver to reduce the risk of over penetration of the osteotomy and implant placement [38].

The limitations of this study were: a small number of cases; saline irrigation was required to limit overheating and deformation of the resin while drilling, and also to remove debris. However, it is possible that remaining debris could have prevented correct seating of the implant within the osteotomy, and this could have had an impact both on the seating of the implant and the vertical depth and thus may explain the larger comparative deviation from the planned position in Case 2, fully guided. In addition, Cases 1, 2, and 6 would not meet the criteria for in vivo study as the planned distance in all these cases was less than 4 mm, in part because we wanted to use a standard-sized implant (10 mm). In Case 6 in particular, both techniques resulted in an implant placement close to the IAN than the planned position and the cutting tip of the drill would likely have caused damage to the IAN. Bony compression may also have resulted in a crushing injury to the IAN in this case [39].

## 4. Conclusions

This bench study, within its limitations, demonstrated acceptable surgical accuracy for both FH and FG techniques that would allow safe placement of implants close to vital structures such as the IAN when a safety zone of 3 mm is allowed. Nevertheless, a better margin of error was observed for FG surgery concerning the angular deviation and controlling the distance of the implant to the IAN using R2 Gate^®^ system. It can be concluded from this study that FG surgery offers improved surgical accuracy over FH technique. However, surgeons undertaking FG surgery should be familiar with the inherent inaccuracies of drill guide design, manufacturing, and application errors and must have the experience and ability to manage these limitations and be prepared to revert to conventional freehand implant placement techniques. Future research could focus on the time required for the implant placement its impact and the cost-effectiveness of FG surgery which is essential in the decision making for the approach. Further to this, selecting the large number of sample size collected from multiple centres with techniques used by different clinicians will help in gauging the consistency of results drawn from this pilot study.

## Figures and Tables

**Table 1:** Description of the selected cases categories. Abbreviations: lower left (LL), lower right (LR), inferior alveolar nerve (IAN).

Case Number	Selected Cases Description
Case 1	Missing LR first molar, bound saddle, socket preservation, IAN inferior
Case 2	Missing LR first molar, bound saddle, socket preservation, IAN inferolateral
Case 3	Missing LR first molar, socket preservation, IAN inferior
Case 4	Missing LL second molar, socket preservation, IAN inferior, LL first molar also missing, larger guide span between bound saddles
Case 5	Missing LR first molar, bound saddle, socket preservation, IAN inferolateral, laterally inclined lingual bone
Case 6	Missing LL first molar, bound saddle, socket preservation, no lingual fossa, IAN inferolateral, and close to the planned position

**Table 2:** Deviation from the planned position for freehand surgery. Positive values of deviation indicated a surgical position further away from the IAN, negative values of deviation indicate a surgical position closer to the IAN.

Free Hand	Planned Distance to IAN (mm)	Distance to IAN (mm)	Deviation (mm)	Coronal Deviation (mm)	Apical Deviation (mm)	Angular Deviation (°)
1	3.21	4.28	+1.07	1.00	2.02	8.00
2	2.50	2.79	+0.29	0.87	2.17	9.12
3	6.29	7.91	+1.62	1.88	2.03	1.99
4	4.70	4.93	+0.23	0.70	0.93	4.88
5	4.18	6.73	+2.55	1.69	3.38	11.78
6	1.29	1.22	−0.07	0.44	0.78	2.82
Mean	3.70 ± 1.76	4.64 ± 2.47	0.95 ± 1.00	1.10 ± 0.57	1.88 ± 0.95	6.43 ± 3.82

**Table 3:** Deviation from the planned position for fully guided surgery. Positive values of deviation indicated a surgical position further away from the IAN, negative values of deviation indicate a surgical position closer to the IAN.

Fully Guided	Planned Distance to IAN (mm)	Distance to IAN (mm)	Deviation (mm)	Coronal Deviation (mm)	Apical Deviation (mm)	Angular Deviation (°)
1	3.21	2.96	−0.25	0.43	0.53	0.97
2	2.50	3.13	+ 0.63	0.79	0.92	0.98
3	6.29	6.33	+ 0.04	0.14	0.14	0.16
4	4.70	4.80	+ 0.10	0.22	0.22	0.95
5	4.18	4.35	+ 0.17	0.21	0.21	0.97
6	1.29	1.08	−0.21	0.29	0.29	0.69
Mean	3.70 ± 1.76	3.78 ± 1.80	0.08 ± 0.32	0.35 ± 0.24	0.43 ± 0.27	0.78 ± 0.33

**Table 4:** Comparing the deviation of planned to placed implant position using free-hand and fully guided techniques.

	Mean Deviation from Planned Position	
Study	Free-Hand	Guided
Fürhauser et al. (2015) [30]	-	Entry 0.8 mm Apex 1.2 mm Angle 2.7°
Schnutenhaus et al. (2016) [31]	-	Entry 1.0 mm Apex 1.6 mm Angle 5.0°
Tahmaseb et al. (2018) [29]	-	Entry 0.9 mm Apex 1.2 mm Angle 3.3°
Vercruyssen et al. (2015) [32]	Entry 2.8 mm Apex 3.1 mm Angle 9.1°	Entry 1.4 mm Apex 1.6 mm Angle 3.0°
Younes et al. (2018) [33]	Entry 1.5 mm Apex 2.1 mm Angle 7.0°	Entry 0.7 mm Apex 1.0 mm Angle 2.3°
Mistry et al. (2021)	Entry 1.1 mm Apex 1.9 mm Angle 6.4°	Entry 0.4 mm Apex 0.4 mm Angle 0.8°

## Data Availability

Not applicable.

## References

[1] Buser D., Arx T. (2000). Surgical procedures in partially edentulous patients with ITI implants. Clin. Oral Implant. Res..

[2] Renton T. (2010). Prevention of Iatrogenic Inferior Alveolar Nerve Injuries in Relation to Dental Procedures. Dent. Update.

[3] Yilmaz Z., Ucer C., Scher E., Suzuki J., Renton T. (2016). A Survey of the Opinion and Experience of UK Dentists. Implant. Dent..

[4] Lin C.-C., Wu C.-Z., Huang M.-S., Huang C.-F., Cheng H.-C., Wang D.P. (2020). Fully Digital Workflow for Planning Static Guided Implant Surgery: A Prospective Accuracy Study. J. Clin. Med..

[5] De Angelis P., Manicone P.F., De De Angelis S., Grippaudo C., Gasparini G., Liguori M.G., Camodeca F., Piccirillo G.B., DeSantis V., D’Amato G. (2020). Patient and Operator Centered Outcomes in Implant Dentistry: Comparison between Fully Digital and Conventional Workflow for Single Crown and Three-Unit Fixed-Bridge. Materials.

[6] Walker-Finch K., Ucer C. (2020). Five-year survival rates for implants placed using digitally-designed static surgical guides: A systematic review. Br. J. Oral Maxillofac. Surg..

[7] Monje A., Insua A., Wang H.-L. (2019). Understanding Peri-Implantitis as a Plaque-Associated and Site-Specific Entity: On the Local Predisposing Factors. J. Clin. Med..

[8] Pauwels R., Jacobs R., Singer S., Mupparapu M. (2015). CBCT-based bone quality assessment: Are Hounsfield units applicable?. Dentomaxillofac. Radiol..

[9] Beuer F., Schweiger J., Edelhoff D. (2008). Digital dentistry: An overview of recent developments for CAD/CAM generated restorations. Br. Dent. J..

[10] Van Noort R. (2012). The future of dental devices is digital. Dent. Mater..

[11] Greenstein G., Tarnow D. (2006). The Mental Foramen and Nerve: Clinical and Anatomical Factors Related to Dental Implant Placement: A Literature Review. J. Periodontol..

[12] Wareing J., Singh N., Jaffa N., Yankova Z., Cox B., McCaul J. (2016). The role of CBCT in OMFS practice. Br. J. Oral Maxillofac. Surg..

[13] NowakA R., ZawiślakB E., BatyckiB J. (2014). Factors affecting for the injury of lingual and inferior alveolar nerve during third lower molar surgery in the mandible. Dent. Med. Probl..

[14] Eggert F.-M., Levin L. (2018). Biology of teeth and implants: The external environment, biology of structures, and clinical aspects. Quintessence Int..

[15] McAllister P., Watson M., Burke E. (2017). A Cost-Effective, In-House, Positioning and Cutting Guide System for Orthognathic Surgery. J. Maxillofac. Oral Surg..

[16] Parnia F., Yazdani J., Javaherzadeh V., Dizaj S.M. (2017). Overview of Nanoparticle Coating of Dental Implants for Enhanced Osseointegration and Antimicrobial Purposes. J. Pharm. Pharm. Sci..

[17] Tarnow D., Cho S.-C., Wallace S. (2000). The Effect of Inter-Implant Distance on the Height of Inter-Implant Bone Crest. J. Periodontol..

[18] Grunder U., Gracis S., Capelli M. (2005). Influence of the 3-D bone-to-implant relationship on esthetics. Int. J. Periodontics Restor. Dent..

[19] Burke R., Richardsen A. (2019). Increasing Occupational Health and Safety in Workplaces. Increasing Occupational Health and Safety in Workplaces.

[20] Smitkarn P., Subbalekha K., Mattheos N., Pimkhaokham A. (2019). The accuracy of single-tooth implants placed using fully digital-guided surgery and freehand implant surgery. J. Clin. Periodontol..

[21] Jacobs R., Salmon B., Codari M., Hassan B., Bornstein M.M. (2018). Cone beam computed tomography in implant dentistry: Recommendations for clinical use. BMC Oral Health.

[22] Jang D., Son K., Lee K.-B. (2019). A Comparative Study of the Fitness and Trueness of a Three-Unit Fixed Dental Prosthesis Fabricated Using Two Digital Workflows. Appl. Sci..

[23] Kim S.-M., Son K., Kim D.-Y., Lee K.-B. (2019). Digital Evaluation of the Accuracy of Computer-Guided Dental Implant Placement: An In Vitro Study. Appl. Sci..

[24] Huang L., Zhang X., Mo A. (2021). A Retrospective Study on the Transferring Accuracy of a Fully Guided Digital Template in the Anterior Zone. Materials.

[25] Kühl S., Zürcher S., Mahid T., Müller-Gerbl M., Filippi A., Cattin P. (2012). Accuracy of full guided vs. half-guided implant surgery. Clin. Oral Implant. Res..

[26] Scherer U., Stoetzer M., Ruecker M., Gellrich N.-C., Von See C. (2015). Template-guided vs. non-guided drilling in site preparation of dental implants. Clin. Oral Investig..

[27] Gluckman H., Du Toit J., Salama M. (2015). Guided bone regeneration of a fenestration complication at immediate implant placement simultaneous to the socket-shield technique. Int. Dent..

[28] Davidowitz G., Kotick P.G. (2011). The Use of CAD/CAM in Dentistry. Dent. Clin. N. Am..

[29] Tahmaseb A., Wu V., Wismeijer D., Coucke W., Evans C. (2018). The accuracy of static computer-aided implant surgery: A systematic review and meta-analysis. Clin. Oral Implant. Res..

[30] Fürhauser R., Mailath-Pokorny G., Haas R., Busenlechner D., Watzek G., Pommer B. (2015). Esthetics of Flapless Single-Tooth Implants in the Anterior Maxilla Using Guided Surgery: Association of Three-Dimensional Accuracy and Pink Esthetic Score. Clin. Implant. Dent. Relat. Res..

[31] Schnutenhaus S., Edelmann C., Rudolph H., Luthardt R.G. (2016). Retrospective study to determine the accuracy of template-guided implant placement using a novel nonradiologic evaluation method. Oral Surg. Oral Med. Oral Pathol. Oral Radiol..

[32] Vercruyssen M., Coucke W., Naert I., Jacobs R., Teughels W., Quirynen M. (2014). Depth and lateral deviations in guided implant surgery: An RCT comparing guided surgery with mental navigation or the use of a pilot-drill template. Clin. Oral Implant. Res..

[33] Younes F., Cosyn J., De Bruyckere T., Cleymaet R., Bouckaert E., Eghbali A. (2018). A randomized controlled study on the accuracy of free-handed, pilot-drill guided and fully guided implant surgery in partially edentulous patients. J. Clin. Periodontol..

[34] Baab D.A., Ammons W.F., Selipsky H. (1977). Blood Loss During Periodontal Flap Surgery. J. Periodontol..

[35] Vasconcelos J.D.A., Avila G.B., Ribeiro J.C., Dias S.C., Pereira L.J. (2008). Inferior alveolar nerve transposition with involvement of the mental foramen for implant placement. Med. Oral Patol. Oral Y Cir. Bucal.

[36] Garcés M.A.S., Escoda-Francolí J., Gay-Escoda C. (2011). Implant Complications. Implant Dentistry—The Most Promising Discipline of Dentistry.

[37] Hegedus F., Diecidue R.J. (2006). Trigeminal nerve injuries after mandibular implant placement—Practical knowledge for clinicians. Int. J. Oral Maxillofac. Implant..

[38] Vercruyssen M., Laleman I., Jacobs R., Quirynen M. (2015). Computer-supported implant planning and guided surgery: A narrative review. Clin. Oral Implant. Res..

[39] Misch C.E., Resnik R. (2010). Mandibular Nerve Neurosensory Impairment After Dental Implant Surgery: Management and Protocol. Implant. Dent..

